# Women's empowerment and current contraceptive use in Pakistan: informed by theory of gender and power

**DOI:** 10.3389/fgwh.2024.1360052

**Published:** 2024-11-21

**Authors:** Bhavita Kumari, Mai Do, Aubrey Spriggs Madkour, Janna Marie Wisniewski

**Affiliations:** ^1^ Global Community Health and Behavioral Sciences Department, Tulane University, New Orleans, LA, United States; ^2^ Health Policy and Management Department, Tulane University, New Orleans, LA, United States

**Keywords:** contraceptive use, women's empowerment, household power dynamics, intimate partner violence, Pakistan

## Abstract

**Introduction:**

It is evident from the stagnant modern contraceptive rate and the growing population of Pakistan that the family planning (FP) programs in Pakistan have failed to deliver successfully. The study examines the association of domains of women's empowerment, following the Theory of Gender and Power, with the current use of contraceptive methods and how intimate partner violence (IPV) can moderate such associations in Pakistan.

**Methods:**

Married women of reproductive age from the Pakistan DHS (2017–18) were included in the analysis (*n* = 14,502). Key independent variables were identified using Connell's Theory of Gender and Power operationalized by Wingood and DiClemente, and constructs were created using principal component analysis. Multinomial logistic regressions were conducted to assess the relationships of the three empowerment divisions (i.e., sexual division of labor, sexual division of power, and cathexis), to the current use of contraceptives.

**Results:**

When all empowerment domains were included in the model along with covariates, education (sexual division of labor), sex negotiations (sexual division of power), and husband's fertility intentions (cathexis) remained significant in their associations with modern contraceptive use.

**Conclusion:**

This is the first study in Pakistan to examine multi-faceted empowerment, applying Connell's theory of gender and power to identify key domains associated with contraceptive use. A multi-prong approach to FP programs that aims to improve specific domains of women's empowerment and to increase FP service use may be more likely to succeed than stand-alone programs.

## Introduction

The inclusion of women's empowerment in the SDGs highlights the importance of empowerment for development and health (1). Empowerment may contribute to women's positive sexual and reproductive health outcomes, including better pregnancy outcomes and increased family planning (FP) practices. Women's empowerment is a complex concept that has been defined and conceptualized by various researchers differently. Often, socio-demographic characteristics like education and employment status have frequently been used as proxies (2), depending on the local context and the dimension of empowerment being explored (3). However, they fail to explain women's empowerment adequately as a phenomenon. Kabeer conceptualized empowerment as the process of agency, resources, and achievements, rather than viewing it as a status, “by which those who have been denied the ability to make strategic life choices acquire such an ability” (4). Measuring women's empowerment has been a challenge, partly due to variations in its definitions and secondly, that it is a multidimensional process with the interrelation among different levels depending on social context. This study will overcome the limitations mentioned above by incorporating multidimensional measures of women's empowerment at the individual, couple, and societal levels, employing the Theory of Gender and Power (5), one of the theories that present women's empowerment as a comprehensive concept.

Globally, the number of women requiring family planning grew from 0.7 billion in 1990 to 1.1 billion in 2021, a 62% increase (6). This need is being increasingly met through modern contraceptive methods. Concurrently, the global total fertility rate declined from 3.3 births per woman in 1990 to 2.3 births per woman in 2021 (6). The number of women using modern contraception nearly doubled from 35% (467 million) in 1990 to 45% (874 million) in 2021 (6). The number of women of reproductive age using traditional contraceptive methods rose from 84 million in 1990 to 92 million in 2021, though their proportion decreased from 6% to 5% (6). However, the modern contraceptive rate is still below 50% in 41 countries, mostly in low-to middle- income countries (6).

Pakistan's CPR (34%) is much lower than its neighboring countries, such as 56% in India and 62% in Bangladesh (7–9); modern contraceptive prevalence has remained consistently low over the last five years: 26% in 2012–13, and 25% in 2017–18 (7). The Pakistan Demographic and Health Survey (PDHS) 2017–18 reported that about half (51%) of women in the country want to delay pregnancy or want no more children (7). FP was introduced and implemented through 5-year plans in Pakistan since the 1950s (10). Pakistan committed to the ICPD's POA in 1994, London Summit on Family Planning in 2012, and implemented Costed Implementation Plans to increase contraceptive uptake in Pakistan (10). Despite being one of the earliest FP planning programs in the world and being a signatory to international FP commitments, Pakistan's contraceptive prevalence rate (CPR) has been stagnant since 1990 (7).

FP programs in Pakistan have not yet succeeded in lowering the fertility rate. There is a pressing need to investigate how to improve the success of FP programs in the country; one of many ways to do so is to understand the roles of women's empowerment in contraceptive use. While Pakistan has socio-cultural contexts similar to that of India and Bangladesh, Pakistan has a history of political instability, terrorism, and security concerns (11). These conflicts, along with the gendered social norms such as household roles, women's status and safety in the society, contribute to limiting the empowerment of Pakistani women (12). Therefore, it is crucial to understand factors associated explicitly with the low uptake of contraceptives in Pakistan. Southern Asia has reached a gender parity score of 63.4%, the second lowest among the eight regions described by the Global Gender Gap Report (13). While Bangladesh and India ranked 59th and 127th for gender equity, Pakistan ranked 142nd out of 146 countries (13).

It is crucial to understand that gender equity is positively associated with women's health behaviors in previous research in varied settings (14). There is abundant evidence of the associations between women's empowerment and contraceptive use. Blanc described a significant association between power balance in sexual relationships and reproductive behaviors in multiple studies (5, 15). Woman's decision-making power and attitudes toward intimate partner violence (IPV) were also important determinants of uptake of reproductive health services (16–18). Women with more decision-making power were more likely to visit the healthcare facility, increasing physical access to contraceptive methods, compared to women with less decision-making power (17).

Women's increased participation in higher levels of education and greater economic opportunities likely provide them with more bargaining power and decision-making authority within the household (19, 20). While Shakya reported no associations between women's empowerment and couple's discordance on family size preference in India (21), she also reported when wives had more education, it was unlikely that the wife would prefer more children than the husband. A qualitative study among women with a history of IPV generated themes of reproductive control, highlighting women's lack of negotiating power in contraceptive use (22).

Most studies have only focused on the relationship of contraceptive use with proxies that contribute to women’s empowerment (4, 23). Other studies which explained contraceptive use by empowerment measures employed single or limited constructs of women’s empowerment (18, 24, 25). Similarly, most studies in Pakistan have focused on either proxies or single factors which contribute to women’s empowerment, that is, woman’s age, education, decision-making and household wealth index (19, 26). A study in Punjab, Pakistan found a woman’s age, education, and household wealth index positively and significantly associated with contraception use (26). A national survey in Pakistan in 2000 also reported decision autonomy and social norms to be positively associated with contraceptive use, independent of economic development in Pakistan (19).

Connell's theory can, therefore, be employed to comprehensively operationalize the various indicators of women's empowerment and assess their associations with contraceptive use in Pakistan. The theory was, hence, applied in the study to investigate the association between various women's empowerment measures and contraceptive use in Pakistan. The research objective of this study was to examine the association between the current use of contraceptives and women's empowerment among married women of reproductive age in Pakistan

### Conceptual framework

This study was guided by Connell's Theory of Gender and Power, as operationalized by Wingood and DiClemente, since it explicitly incorporates gender dynamics at multiple levels and its influence on women's health outcomes (5). It applies different constructs of women's empowerment, including household decision-making, attitudes toward IPV, economic autonomy, and gender norms, toward reproductive behaviors. The Theory of Gender and Power has been employed in studies examining the relationship between gender inequality and HIV risk behaviors in Africa (1, 27). The theory conceptualizes three social structures that affect gender relations or *domains*: sexual division of labor, sexual division of power, and the structure of cathexis (5).

The “*sexual division of labor*” implies that unequal financial opportunities for women lead to economic inequities at all institutional levels; the construct builds upon factors such as living below the poverty line, homelessness, under-employment, low education, no health insurance, high demand/low control work, and being an ethnic minority or underaged. Economic equities have been studied to have positive association with women's health and healthcare access (17, 28). One intervention in Pakistan reported a positive association between financial independence via micro-credit and women's empowerment (29).

The “*sexual division of power*” implies that imbalance of decision-making control can result in inequities in power in relationships, and access to the health system; the constructs build upon experiences and attitudes towards sexual or physical abuse, household decision-making, and difficulties accessing health services due to cultural barriers. A literature review found significantly positive relationships between empowerment and current use of contraception, where empowerment was measured by two variables, that is household decision-making and mobility (30).

“*Cathexis*” implies to the affective aspects of relationships including the gender biases in the gender roles and gendered societal norms; the constructs build upon multiple social constructs including religious restrictions, conservative beliefs, women's say in choosing a partner, *watta satta* (bride exchange marriages)^1^, having an older partner, mistrust in the health system or limited and knowledge. Peer pressure, the influence of opinionated leaders, religious beliefs, and family acceptance of contraceptive use are hypothesized to influence women's sexuality and contraceptive use by creating a taboo in developing countries (31).

## Methods

This cross-sectional study utilized the women's data of the PDHS 2017–18. The survey was conducted in Pakistan's urban and rural areas in 2017–18, employing a two-stage stratified sampling design. Clusters, (i.e., census enumeration blocks, were selected in the first stage, and households within each cluster were selected systematically in the second stage (7). We included women who were aged between 15 and 49 and married at the time of the PDHS 2017–18 survey (*N* = 14,502). Widowed, divorced, and separated women were excluded as the study focuses on women's empowerment within a couple and household context. The key outcome variable was the current use of contraceptives, categorized by modern and traditional methods. Traditional methods included periodic abstinence, withdrawal, lactational amenorrhea, and other folkloric methods. Modern contraceptives included oral contraceptive pills, emergency pills, IUDs, injectable contraceptives, implants, standard days methods, male condoms, and permanent contraceptives, including tubal ligation and vasectomy.

**Figure 1:**
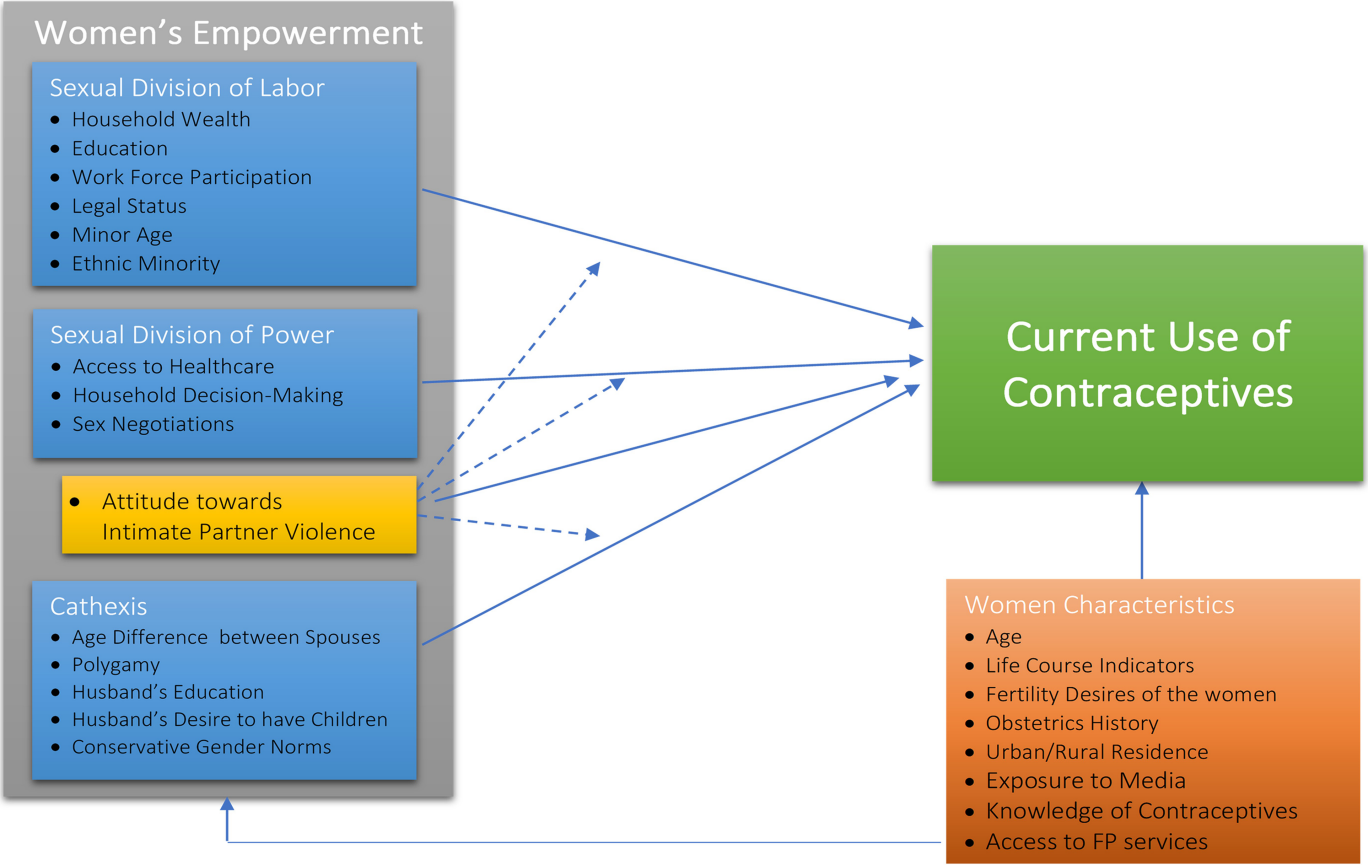
Conceptual framework of relationships between women's empowerment and women characteristics and current use of contraceptives among married women in Pakistan.

Women's empowerment factors were identified using Connell's Theory of Gender and Power as operationalized by Wingood and DiClemente (5), grouped into three domains: sexual division of labor, sexual division of power, and cathexis, as illustrated in Figure 1 and Table 1. Empowerment variables were recoded to dichotomous scales such that a higher score indicates higher empowerment. The individual measures of women's empowerment were used to construct latent measures of domains of empowerment, using principal component analysis, along with the Cronbach's alpha and eigenvalues for the given constructs. The constructs, initially generated as continuous variables, were later recoded into categorical variables as described in Table 1. Further, sociodemographic variables were entered in the model to control for factors outside of women's empowerment that can potentially influence the outcome.

**Table 1:** Principal component analysis of items included in empowerment constructs as measured in Pakistan DHS 2017–18. (*N* = 14,502).

	Variable	Alpha Cronbach (Eigenvalue)	Questions/items	Factor loading
Sexual division of power	Household decision-making	0.88 (2.96)	Who usually decides how your husband's earnings will be used?	0.50
			Who usually makes decisions about making major household purchases?	0.52
			Who usually decides about healthcare for wife?	0.49
			Who usually makes decisions about visits to your family or relatives?	0.49
	Attitudes toward IPV	0.90 (3.59)	Is beating justified if wife goes out without telling husband?	0.46
			Is beating justified if wife neglects the children?	0.45
			Is beating justified if wife agues with husband?	0.47
			Is beating justified if wife refuses to have sex with husband?	0.45
			Is beating justified if wife burns the food?	0.41
	Access to healthcare	0.78 (2.42)	Is getting permission to go to the doctor a big problem?	0.51
			Is getting money needed for treatment a big problem?	0.51
			Is the distance to the health facility a big problem?	0.52
			Is wanting to go alone a big problem?	0.46
	Sex negotiations	0.71 (1.55)	Can you say no to your husband if you do not want to have sexual intercourse?	0.71
			Could you ask your husband to use a condom if you wanted him to?	0.71

Analysis was conducted using Stata 12 statistical software (32), using -*svy* set of commands to account for the complex two-stage cluster sampling design of the DHS. Correlation analyses were conducted between potentially correlated variables. Multinomial logistic regressions were conducted to assess the relationship of the three empowerment divisions, individually and then all together, with current use of contraceptives. Relative risk ratios were used to compare the current use of modern contraceptives and the current use of traditional methods users against non-use of any contraceptives. A *p*-value of 0.05 was considered statistically significant.

## Results

The study sample consisted of almost two-thirds of women being non-users, one-fourth being modern contraceptive users, and nearly 10% being traditional method users. The demographic characteristics of the sample are reported in Supplementary Table S1.

### Bivariate analysis

Table 2 reports the bivariate analysis and chi-square tests of independence conducted to assess significant differences of reported empowerment and covariates according to current contraceptive use. Among the women who had no education, 7.3% use traditional methods and 21.3% use modern methods. Among the women who had primary or more education, 11.3% use traditional methods and 28.2% use modern methods. Similarly, among the women who had no land, 9.1% use traditional methods and 24.6% use modern methods. Among the women who owned land, 14.2% use traditional methods and 31.0% use modern methods. Similarly, other variables are also analyzed too.

**Table 2:** Bivariate results of the association between sample characteristics and current contraceptive use status, Pakistan DHS 2017–18 (*N* = 14,502).

			Non-user 65.8% (*n* = 9,786)	Traditional methods users 9.3% (*n* = 1,311)	Modern methods users 24.8% (*n* = 3,405)	
		Characteristics	Row% (*n*)	Row% (*n*)	Row% (*n*)	
Empowerment variables	Sexual division of labor	Wealth				(*p* < 0.01)
		Poorest	79.9 (2,284)	3.2 (95)	16.9 (408)	
		Poorer	71.1 (2,254)	6.5 (206)	22.4 (641)	
		Middle	63.3 (1,851)	10.1 (283)	26.6 (723)	
		Richer	61.6 (1,710)	11.0 (317)	27.4 (736)	
		Richest	55.5 (1,687)	14.9 (410)	29.6 (897)	
		Education				(*p* < 0.01)
		No education	71.4 (5,384)	7.3 (504)	21.3 (1,425)	
		Primary or higher	60.5 (4,402)	11.3 (807)	28.2 (1,980)	
		Work force participation (*n* = 14,499)				(*p* = 0.04)
		No	66.1 (8,443)	9.7 (1,122)	24.3 (2,821)	
		Yes	64.7 (1,340)	7.9 (189)	27.4 (584)	
		Legal status				(*p* = 0.004)
		No	66.3 (8,443)	9.1 (1,122)	24.6 (2,821)	
		Yes	54.9 (1,340)	14.2 (189)	31.0 (584)	
		Minor age				(*p* < 0.01)
		No	65.4 (9,582)	9.4 (1,306)	25.2 (3,398)	
		Yes	95.4 (204)	2.9 (5)	1.7 (7)	
	Sexual division of power	Household decision-making				(*p* < 0.01)
		No decision-making	73.7 (4,199)	7.7 (430)	18.6 (1,027)	
		Moderate decision-making	63.8 (3,013)	9.4 (428)	26.7 (1,216)	
		High decision-making	58.7 (2,574)	11.2 (453)	30.1 (1,162)	
		Attitudes toward IPV				(*p* < 0.01)
		No intolerance	71.7 (2,611)	6.4 (227)	21.9 (708)	
		Moderate intolerance	69.0 (2,466)	8.2 (282)	22.8 (721)	
		High intolerance	62.3 (4,709)	10.9 (802)	26.8 (1,976)	
		Access to healthcare				(*p* < 0.01)
		Big problem	71.9 (3,165)	7.9 (321)	20.2 (776)	
		Moderate problem	68.1 (4,050)	8.2 (485)	23.7 (1,322)	
		No problem	58.3 (2,571)	11.9 (505)	29.8 (1,307)	
		Sex negotiations				(*p* < 0.01)
		No	76.6 (4,407)	6.3 (340)	17.0 (829)	
		Yes	58.8 (5,379)	11.3 (971)	29.9 (2,576)	
	Cathexis	Husband's education (*n* = 14,497)				(*p* < 0.01)
		No education or primary	69.2 (4,240)	7.3 (419)	23.5 (1,270)	
		Secondary or higher	63.0 (5,543)	11.0 (892)	25.9 (2,133)	
		Husband's fertility intentions (*n* = 13,523^a^)				(*p* < 0.01)
		Husband wants more or wife doesn't know	78.8 (5,245)	7.7 (492)	13.6 (866)	
		Husband wants fewer or same as wife	66.7 (4,534)	12.4 (819)	20.9 (1,567)	
		Characteristics^†^	Mean ± SD	Mean ± SD	Mean ± SD	
		Age difference between spouses (*n* = 14,495)	5.2 ± 5.4	5.2 ± 4.8	5.4 ± 4.9	(*p* < 0.01)
		Characteristics	Row% (*n*)	Row% (*n*)	Row% (*n*)	
Control variables		Age				(*p* < 0.01)
		15–24	84.3 (2,456)	4.4 (134)	11.3 (322)	
		25–29+	71.6 (2,240)	7.6 (225)	20.8 (612)	
		30–34	57.8 (1,689)	12.3 (313)	29.9 (772)	
		35–39	55.9 (1,515)	11.7 (294)	32.4 (805)	
		40–49	57.5 (1,886)	11.2 (345)	31.2 (894)	
		Fertility intentions (*n* = 14,490)				(*p* < 0.01)
		Want more/undecided	82.9 (6,465)	5.7 (421)	11.4 (881)	
		Want no more/sterilized/infecund	46.5 (3,314)	13.4 (888)	40.1 (2,521)	
		Residence				(*p* < 0.01)
		Urban	57.5 (4,316)	14.0 (825)	28.6 (1,831)	
		Rural	70.6 (5,470)	6.7 (486)	22.7 (1,574)	
		Knowledge of contraceptives (Yes)	65.2 (9,493)	9.5 (1,307)	25.3 (3,405)	
		Access to FP services (Yes)	58.3 (1,930)	9.7 (349)	32.0 (1,131)	(*p* < 0.01)
		Characteristics^†^	Mean ± SD	Mean ± SD	Mean ± SD	
		Age at marriage	19.3 ± 4.2	19.6 ± 4.0	19.0 ± 3.9	(*p* < 0.01)
		Number of living sons	1.3 ± 1.4	2.0 ± 1.3	2.1 ± 1.3	(*p* < 0.01)

Tabulations account for survey weights. *p*-value based on chi-square test of independence.

^a^ >5% loss of responses.

^†^ *p*-value based on one-way ANOVA.

### Multivariate analysis: individual empowerment domains

**Figure 2:**
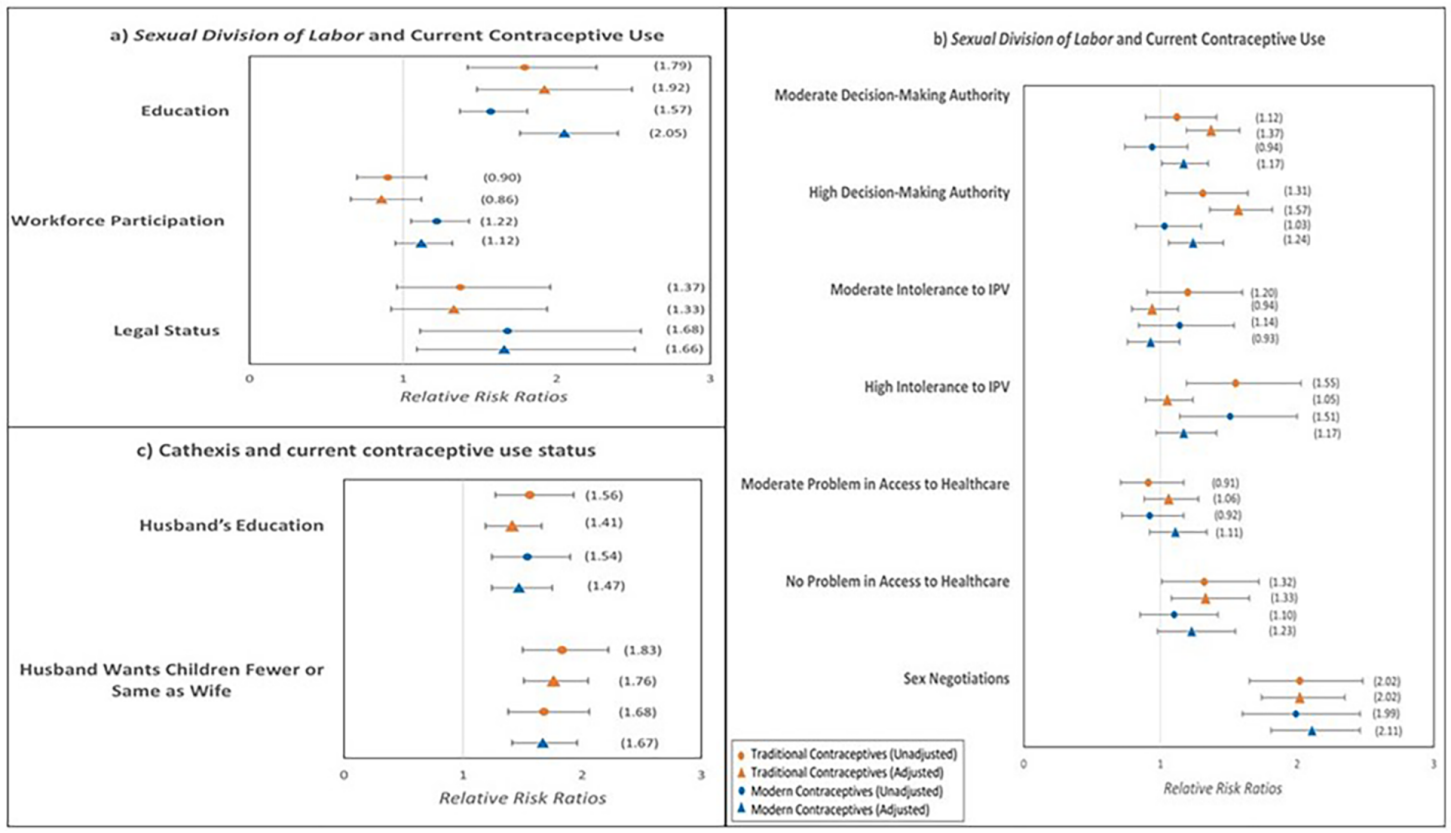
Multinomial logistic regression results of associations between the three domains of women's empowerment and current contraceptive use status (*N* = 14,502).

Figure 2 presents the multinomial logistic regressions results, each graph presenting the associations between contraceptive use and each empowerment domain according to the Theory of Gender and Power using relative risk ratios (RRRs) with unadjusted and adjusted models. For the sexual division of labor, education and legal status remained significant factors influencing contraceptive use. For the sexual division of power, household decision-making, attitudes toward IPV, and sex negotiations remained significant contributors to the current use of contraceptives. For the cathexis, both husband's education and fertility intentions remained significant factors.

### Multivariate analysis: all empowerment domains

Table 3 shows unadjusted (model I) and adjusted (model II) results from the multinomial logistic regressions describing the relationships of the current use of contraceptives with all three empowerment divisions together. Women who had primary or higher education were significantly 1.45 times more likely to use traditional contraceptive methods (95%CI: 1.11, 1.90) and 1.72 times more likely to use modern contraceptive methods (95%CI: 1.43, 2.07), as compared to women with no education after adjusting for covariates. Women who owned property or land were significantly 1.54 times more likely to use traditional contraceptive methods than women with no property or land (95%CI: 1.01, 2.33). Women who had high intolerance toward IPV were significantly 1.33 times more likely to use traditional contraceptives than women who had no intolerance toward IPV (95%CI: 1.01, 1.76). Women who could negotiate sexual activities were significantly 1.79 times more likely to use traditional contraceptive methods (95%CI: 1.43, 2.25) and 2.24 times more likely to use modern contraceptive methods (95%CI: 1.88, 2.68), as compared to women who could not negotiate sexual activities. Women whose husbands wanted fewer children or same as the wife were significantly 0.97 times less likely to use traditional contraceptive methods (95%CI: 0.92, 1.01) and 0.92 times less likely to use modern contraceptive methods (95%CI: 0.88, 0.96), as compared to women whose husband wanted more children than the wife.

**Table 3:** Multinomial logistic regression results for associations between empowerment characteristics and current contraceptive use status, Pakistan DHS 2017–18 (*N* = 14,502) (reference category: no contraceptive use).

		Model I		Model II	
		Traditional methods users 9.3% (*n* = 1,311)	Modern methods users 24.8% (*n* = 3,405)	Traditional methods users 9.3% (*n* = 1,311)	Modern Methods Users 24.8% (*n* = 3,405)
	Characteristics	RRR (95% CI)	RRR (95% CI)	ARRR (95% CI)	ARRR (95% CI)
Sexual division of labor	Education				
	No education	–	–	–	–
	Primary or higher	1.22 (0.96, 1.56)	1.48 (1.25, 1.74)**	1.45 (1.11,1.90)**	1.72 (1.43,2.07)**
	Workforce participation				
	No	–	–	–	–
	Yes	0.85 (0.65, 1.10)	0.95 (0.79, 1.14)	0.86 (0.65,1.13)	0.93 (0.77,1.11)
	Legal status				
	No	–	–	–	–
	Yes	1.50 (0.98, 2.27)	1.29 (0.89, 1.85)	1.54 (1.01,2.33)p	1.37 (0.95,1.99)
Sexual division of power	Household decision-making				
	None	–	–	–	–
	Moderate	1.13 (0.89, 1.43)	1.18 (0.99, 1.40)	0.96 (0.75,1.22)	1.07 (0.90, 1.27)
	High	1.33 (1.05, 1.68)*	1.16 (0.97, 1.39)	1.06 (0.84,1.33)	1.03 (0.85, 1.25)
	Attitudes toward IPV				
	No intolerance	–	–	–	–
	Moderate intolerance	1.15 (0.86, 1.54)	0.95 (0.76, 1.19)	1.09 (0.80, 1.48)	0.91 (0.72, 1.17)
	High intolerance	1.36 (1.04, 1.78)*	0.94 (0.77, 1.16)	1.33 (1.01, 1.76)p	0.99 (0.79, 1.23)
	Access to healthcare				
	Big problem	–	–	–	–
	Moderate problem	0.84 (0.66–1.08)	0.97 (0.79–1.20)	0.85 (0.67, 1.09)	0.98 (0.79, 1.21)
	No problem	1.21 (0.93–1.56)	1.08 (0.84–1.38)	1.00 (0.77, 1.29)	1.03 (0.79, 1.33)
	Sex negotiations				
	No	–	–	–	
	Yes	1.86 (1.49, 2.31)**	2.27 (1.92, 2.68)**	1.79 (1.43, 2.25)**	2.24 (1.88, 2.68)**
Cathexis	Husband's education				
	No education or primary	–	–	–	–
	Secondary or higher	1.11 (0.99, 1.24)	1.06 (0.97, 1.16)	1.12 (0.99, 1.25)	1.09 (0.99, 1.19)
	Husband's fertility intentions				
	Husband wants more or wife does not know	–	–	–	–
	Husband wants fewer or same as wife	0.95 (0.91, 0.99)*	0.91 (0.87, 0.94)**	0.97 (0.92, 1.01)**	0.92 (0.88, 0.96)**

Tabulations account for survey weights. Model II: Model I + covariates (age, number of sons, residence, access to FP). RRR, relative risk ratio; ARRR, adjusted relative risk ratio. *p*-value based on logistic regression.

* *p*-value <0.05.

** *p*-value < 0.05.

## Discussion

It is imperative to identify how gender inequity, empowerment, and IPV may be associated with contraceptive use in Pakistan because of low contraceptive uptake while gender dynamics may be changing. With the increasing participation of women in education and employment, the gender dynamics are slowly transforming at the societal level. Yet there have been limited studies in Pakistan examining women's empowerment with a holistic approach. This study aimed to examine the association between multiple domains of women's empowerment and the current use of traditional and modern methods of contraception in Pakistan. Specifically, the focus was on exploring how women's decision-making authority and attitudes toward IPV were associated with contraceptive use in Pakistan. Secondly, the study investigated how women's decision-making authority and their attitudes toward IPV may interact to influence contraceptive use in Pakistan.

Our evidence supported the hypothesis of the study that a higher level of women's empowerment was associated with an increased likelihood of current use of contraceptives among married women of reproductive age in Pakistan. Significant associations were found between contraceptive use and various factors contributing to the three domains of empowerment. The use of modern methods was associated with sexual division of labor (education, property ownership), sexual division of power (ability to negotiate sexual activity), and cathexis (husband's fertility intentions). The use of traditional methods was further associated with high intolerance toward violence as well. A multi-country study, in which associations between women's empowerment and contraceptive use in Sub-Saharan African countries were examined, also reported positive associations: household decision-making authority, ability to negotiate sexual activity, and intolerance toward IPV were significantly associated with contraceptive use (18). Another multi-level analysis of 32 African countries also reported significantly higher contraceptive use among richest households, working women or women whose partners had secondary or higher education (33). A multi-county analysis in South-east Asian context reported association of contraceptives use with labor force participation. The study also reported association between high decision-making and contraceptive use in Cambodia (34). Similar findings were also reported in Burkino Faso where participation in household decision-making, freedom in accessing healthcare, and opposition to domestic violence were associated with modern contraceptive use (35).

Studies in the neighboring countries of India and Bangladesh reported household decision-making autonomy to be positively associated with modern contraceptive use (36, 37). A study using Pakistan DHS 2012–13 reported women’s economic empowerment to be positively associated with contraceptive use and negatively associated with unintended pregnancies (38). In contrast, one study using Pakistan did not find association between modern contraceptive use and economic empowerment (12), however this study compared use of modern contraceptives with traditional contraceptives unlike our study which compared modern and traditional contraceptives with no use of contraceptives individually.

The study's second hypothesis was that as tolerant attitudes toward IPV decrease, the (expected) positive association between empowerment and contraceptive was likely to get stronger. The results were mixed. This study only found this association at one level: when women report high decision-making authority and moderate intolerance toward IPV, the use of traditional methods increases by almost two folds. No other association was seen between the interaction of household decision-making authority and attitudes toward IPV and contraceptive use. Studies in Bangladesh have reported mixed results for association between household decision-making authority and experiences of violence (20, 39, 40); one study focusing on the Bangladesh Rural Advancement Committee credit and savings program reported more physical abuse among its members (40). There is a possibility that Pakistani women still accept violence as a tool to avoid conflict in a patriarchal society that is transitioning to increase women's participation in education and employment. There is a possibility that men feel threatened with losing control when women are more empowered (41). Also, literature has suggested that when men cannot financially support the family, the risk of women's maltreatment increases (41). Jewkes suggested that empowerment may lead to violence in the initial period; it becomes a protective factor after attainment of a crucial threshold and when gender roles have reversed significantly (41).

### Recommendations

This study fills the gap in the knowledge of associations between women's empowerment and contraceptive use in Pakistan. Our study has identified the key domains associated with FP practices and whether IPV attitudes had modified such relationships or not. Specifically, the study generates findings concerning both wife and the husband, like both spouses’ education, sex negotiations which involve both spouses, and husband's fertility intentions. These findings inform policies and programs in the design of FP programs and services to target couples at risk of non-use of contraception. Similarly, family planning surveys need to be designed focusing on both spouses’ opinions.

Similarly, integrated programs between FP and IPV, screening, and referral services can be designed for women who are vulnerable to IPV. There is a need to think beyond isolated FP services and health education interventions focusing on FP services only; a multi-prong approach that aims to improve specific domains of women's empowerment may be more likely to succeed than stand-alone programs. Evidence from this study indicates that it is essential to redirect the focus of women's health interventions. Future health programs targeting women's health in Pakistan would benefit from the recommendations of this research to focus on more crucial determinants. In order to facilitate progress towards family planning programs, policy should focus on girls’ education, legal property ownership rights, and male involvement in reproductive health decisions.

Further research is required to understand the mechanisms that define the relationship between empowerment and contraceptives use. Empowerment constructs that are important in the local context need to be explored as well. Further, studies need to incorporate men's perspective into contraceptive studies to understand the couples’ decision-making dynamics. Lastly, efforts need to be made to advance research into supply and demand of contraceptive use.

### Strengths and limitations

One of the significant strengths of the DHS is the representativeness of the sample, which allows the generalization of findings to the population of married women of reproductive age in Pakistan. The average response rate is 97.6%; only 3.3% of clusters scattered over all provinces were excluded due to security concerns. There is likely limited access to healthcare/FP services in these clusters with security issues, so the generalization of the findings in those areas may be conditioned on supplies. On the other hand, a key limitation of the DHS data is its cross-sectional nature, so causality cannot be inferred.

The DHS also collects data for most of the empowerment domains mentioned in the Theory of Gender and Power. However, the DHS does not measure a few domains that might be important in Pakistan, like precious metal (gold, silver) and livestock ownership in rural areas. These assets can contribute to the women's empowerment domains, depending on whether owned by the woman or the household. Information about local cultural norms like dowry practices and exchange marriages was also not collected. The more assets a woman brings in her dowry, the greater her status in her in-laws. Finally, while Pakistan is a dominatingly Islamic country, where fertility control is often discouraged by religious leaders (42), no information is available about religious affiliation, which could strongly influence contraceptive practices.

In addition, while information on the husband in this study was incorporated, such information was reported by women. There is a critical need to explore the factors of reproductive behaviors from the perspectives of both spouses. Further, qualitative research can provide a more comprehensive picture of the domains of empowerment, especially domains at the societal level, and how the different domains of empowerment interact with each other and associate with reproductive decisions in Pakistan.

## Conclusion

This is the first study in Pakistan to examine multi-faceted empowerment, applying Connell's theory of gender and power to identify key domains associated with contraceptive use despite the mentioned limitations. The study explored women's empowerment from the women's perspective and how it may be related to reproductive behaviors in a patriarchal society with transitioning women's position in the society. The study found significant associations between various domains of empowerment, including education, sex negotiations and husband's fertility intentions and contraceptive use, informing policies to integrate women's empowerment with women's health interventions. A multi-prong approach to FP programs that aims to improve specific domains of women's empowerment and to increase FP service use may be more likely to succeed than stand-alone programs. Such understanding is crucial to improve FP programs and women's health in Pakistan, especially when two-thirds of women of reproductive age do not use contraceptives. Further research is required to understand the mechanisms that define the relationship between empowerment and contraceptives use. Further, studies need to incorporate men's perspective into contraceptive studies to understand the couples’ decision-making dynamics.

## Data Availability

Publicly available datasets were analyzed in this study. This data can be found here: DHS Pakistan 2017−18.

## Footnotes

^1^Common in Sub-continent – the tradition involving simultaneous marriages of (usually) brother-sister pairs from two households. (Jacoby, Hanan G., and Ghazala Mansuri. ""Watta Satta": Bride Exchange and Women's Welfare in Rural Pakistan." *The American Economic Review* 100, no. 4 (2010): 1804-825. Accessed September 8, 2020. http://www.jstor.org/stable/27871275.)
