# Supplementary material for: Women's empowerment and current contraceptive use in Pakistan: informed by theory of gender and power
Source: Front Glob Womens Health. 2024 Nov 21;5:1360052. doi: 10.3389/fgwh.2024.1360052 (PMC11617574; doi:10.3389/fgwh.2024.1360052)
Supplement: Supplementary file 1 [file Datasheet1.pdf]

Table 1: Description of control variables potentially associated with women's empowerment, as measured in Pakistan DHS 2017-18. (N = 14,502).

Variables	Questions	Categories	% (n)
Age	Age of the respondent – 5-year groups	*15-19*	5.0 (725)
		20-24	15.7 (2,187)
		25-29	21.1 (3,077)
		30-34	19.8 (2,774)
		35-39	17.3 (2,614)
		40-44	11.2 (1,696)
		45-49	9.9 (1,429)
Wealth Index	Composite measure of a household's cumulative living standard - calculated using household's ownership of selected assets.[106]	*Poorest*	18.2 (2,787)
		Poorer	19.4 (3,101)
		Middle	20.4 (2,857)
		Richer	20.9 (2,763)
		Richest	21.1 (2,994)
Education	What is the highest level of school you attended: primary, secondary, or higher?	*No Education*	48.8 (7,313)
		Primary	16.5 (2,022)
		Secondary or Higher	34.8 (5,167)
Residence	Type of residence where the respondent is currently residing	*Urban*	36.8 (6,972)
		Rural	63.2 (7,530)
Knowledge of Contraceptives	Have you heard of any contraceptive methods?	*Yes*	98.2 (14,205)
Access to FP Services	Did any fieldworker or staff at the health facility speak to you about FP methods?	*Yes*	23.5 (3,410)
Current Contraceptive Use Status	Are you currently using a method to prevent pregnancy? If yes, what method are you using?	*Non-users*	65.8 (9,786)
		Traditional Methods Users	9.3 (1,311)
		Modern Methods Users	24.8 (3,405)
	**Continuous Variables***		**Mean ± SD**
Life Course Indicators	Age at Marriage		19.3 ± 4.1
	Age at First Birth (n=12,630)**		21.1 ± 4.2

Obstetric History	Number of Children Ever Born	3.4 ± 2.5
	Number of Living Sons	1.6 ± 1.4

Tabulations account for survey weights **>5% loss of responses
